# GPx3 knockdown inhibits the proliferation and DNA synthesis and enhances the early apoptosis of human spermatogonial stem cells via mediating CXCL10 and cyclin B1

**DOI:** 10.3389/fcell.2023.1213684

**Published:** 2023-07-07

**Authors:** Si Wu, Zixin Cheng, Ye Peng, Ying Cao, Zuping He

**Affiliations:** ^1^ The Key Laboratory of Model Animals and Stem Cell Biology in Hunan Province, Hunan Normal University School of Medicine, Changsha, China; ^2^ The Research Center of Reproduction and Translational Medicine of Hunan Province, The Manufacture-Based Learning and Research Demonstration Center for Human Reproductive Health New Technology of Hunan Normal University, Changsha, Hunan, China

**Keywords:** GPx3, CXCL10, cyclin B1, human spermatogonial stem cells, proliferation, apoptosis

## Abstract

Spermatogenesis is regulated by genetic and epigenetic factors. However, the genes and signaling pathways mediating human spermatogenesis remain largely unknown. Here, we have for the first time explored the expression, function, and mechanism of glutathione peroxidase 3 (GPx3) in controlling the proliferation and apoptosis of human spermatogonial stem cells (SSCs). We found that GPx3 was expressed in human SSCs. Notably, we revealed that GPx3 knockdown resulted in the decrease in the proliferation, DNA synthesis, and cyclin B1 level in human SSC lines, which possessed the phenotypic features of human primary SSCs. Flow cytometry and TUNEL assays showed that GPx3 silencing led to enhancement of early apoptosis of human SSC line. RNA sequencing was utilized to identify CXCL10 as a target of GPx3 in human SSCs, and notably, both double immunostaining and co-immunoprecipitation (co-IP) demonstrated that there was an association between GPx3 and CXCL10 in these cells. CXCL10-shRNA resulted in the reduction in the proliferation and DNA synthesis of human SSC line and an increase in apoptosis of these cells. Taken together, these results implicate that GPx3 regulates the proliferation, DNA synthesis, and early apoptosis of human SSC line via mediating CXCL10 and cyclin B1. This study, thus, offers a novel insight into the molecular mechanism regulating the fate determinations of human SSCs and human spermatogenesis.

## Introduction

Infertility is one cause for low population growth, which affects approximately 16% of couples worldwide, and 50% of these cases are attributed to male factor infertility (Agarwal et al., 2015). It has been estimated that at least 30 million men are infertile around the world. Azoospermia is the most severe form of male infertility and can be classified into obstructive azoospermia (OA) and non-obstructive azoospermia (NOA). Impaired spermatogenesis is the major reason for NOA, which leads to male infertility (Tharakan et al., 2021). Spermatogenesis is the process by which male germ cells develop from spermatogonial stem cells (SSCs) to become mature spermatozoa, and it is regulated by multiple factors, including epigenetic (Li et al., 2022), genetic (Riera-Escamilla et al., 2022), hormonal (Desai et al., 2022; Liu et al., 2022) and biological clocks (Xie et al., 2022). SSCs are the basis for maintaining normal spermatogenesis and male fertility. In addition, SSCs are one of the most important adult stem cells since they have great plasticity and significance in reproductive and regenerative medicine. We and our peers have reported that SSCs can be directly transdifferentiated *in vitro* into other types of functional cells, e.g., mature hepatocytes (Zhang et al., 2013; Chen et al., 2016; Chen et al., 2017); neurons (Yang et al., 2015); prostate, uterine, and skin epithelial cells (Simon et al., 2009); and haploid spermatocytes with fertilization and developmental potential (Yang et al., 2014). Recently, our team used matrigel to establish a three-dimensional culture system and employed SCF, BMP4, RA, and hormones as the conditioned medium, which induces the differentiation of human SSCs into functional haploid spermatocytes *in vitro* (Sun et al., 2018). As such, studies on the roles and mechanisms of specific genes mediating the self-renewal and apoptosis of human SSCs provide new insights into the pathogenesis of male infertility and contribute to reproductive and regenerative medicine.

The fate determinations of SSCs *in vivo* include self-renewal, differentiation, and apoptosis, which are driven by the precise control of genetic and epigenetic factors. Some progress has been made in uncovering the molecular mechanisms of self-renewal and differentiation of rodent SSCs. Nevertheless, little is known about the genes mediating the fate decisions of human SSCs, probably due to the low proportion of human SSCs in male germ cells (∼0.03%) and the limited source of human testicular tissues (Tegelenbosch and de Rooij, 1993). Therefore, in this study, we used the first human SSC line with unlimited proliferation capacity as previously established by our group, which could provide an adequate source of human SSCs to explore the regulatory mechanisms controlling the fate decisions of human SSCs (Hou et al., 2015). In our previous studies, *FOXP3*, *PAK1*, *TCF3*, and *RNF144B* in association with human SSC line have been shown to regulate human SSC proliferation and apoptosis (Schwaab et al., 1998; Fu et al., 2018; Qiu et al., 2019; Chen et al., 2020; Zhou et al., 2021; Du et al., 2022). Meanwhile, MKK7, FOXP4, SPOC, and ASB9 have been reported to be involved in the regulation of proliferation and apoptosis of human SSCs (Huang et al., 2021; Zhou et al., 2022; Li et al., 2023; Luo et al., 2023). However, it is imperative to identify novel genes required for human SSC fate decisions.

Glutathione peroxidase 3 (GPx3) is a member of the glutathione peroxidase family and an androgen-dependent cytoplasmic enzyme. GPx3 has been examined in a variety of tumors and stem cells due to its significant roles in redox processes and regulation of a variety of signaling molecules. Unlike GPx5, the expression of GPx3 in the epididymis is not specific, and it can be detected in multiple tissues (Schwaab et al., 1998). We have previously shown that EGF increases PAK1 expression in the human SSC line, while PAK1 knockdown reduces proliferation and DNA synthesis and increases apoptosis of the human SSC line (Fu et al., 2018). Notably, we have revealed that PAK1 regulates *GPx3* in the human SSC line (Fu et al., 2018). We further demonstrated that *PAK1* knockdown results in reduction of GPx3 in the human SSC line at transcriptional and translational levels, which reflects the essential role of GPx3 in the regulation of human SSC fate decisions. In this study, we have demonstrated that GPx3 silencing led to a reduction in proliferation, DNA synthesis, and enhancement of early apoptosis in the human SSC line via mediating CXCL10 and cyclin B1. This study thus provides a novel molecular mechanism for controlling human SSC fate determinations.

## Results

### GPx3 is expressed in human spermatogonial stem cells (SSCs)

**FIGURE 1:**
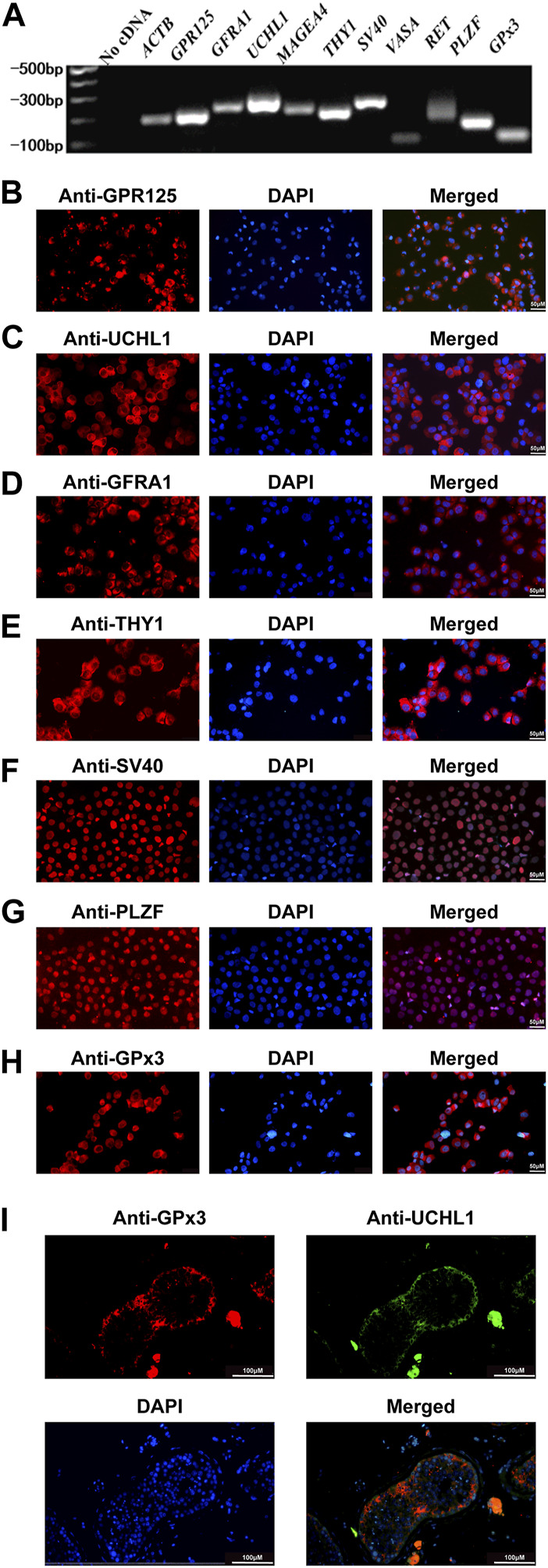
Identification of the human SSC line and the expression of GPx3 in the human SSC line and human testicular tissues: **(A)** RT-PCR revealed that numerous markers of male germ cells and SSCs, including *GPR125*, *GFRA1*, *UCHL1*, *THY1*, *MAGEA4*, *VASA*, *RET*, and *PLZF*, were expressed in the human SSC line. *ACTB* served as a loading control of the total RNA, while water with RT and PCR was used as a negative control. **(B–H)** Immunocytochemical analysis displayed the expression of the special proteins including GPR125 **(B)**, UCHL1 **(C)**, GFRA1 **(D)**, THY1 **(E)**, SV40 **(F)**, PLZF **(G)**, and GPx3 **(H),** in the human SSC line. Scale bars in B–H = 50 μm. **(I)** Immunohistochemistry showed the cellular location of GPx3 in human testicular tissues. UCHL1 was utilized as a marker for human SSCs in human testicular tissues. Scale bars in I = 100 μm.

We have previously found that PAK1 plays an essential role in regulating the proliferation and apoptosis of human SSCs. Using RNA sequencing and qPCR, we demonstrated that the level of GPx3 was decreased by PAK1 silencing. Thus, we speculated that GPx3 is required for the proliferation and apoptosis of human SSCs. To detect the cellular localization of GPx3 in the human SSC line and human testicular tissue, RT-PCR, immunocytochemistry, and immunohistochemistry were applied. The identity of the human SSC line was verified by mRNA and protein expression of numerous markers for human SSCs, including GPR125, GFRA1, UCHL1, MAGEA4, THY1, and PLZF (Figures 1A–G). RT-PCR and immunocytochemistry further displayed that *GPx3* mRNA (Figure 1A) and GPx3 protein (Figure 1H) were detected in the human SSC line. Cell localization of GPx3 in the human testis was determined by double immunostaining using antibodies against GPx3 and UCHL1. We revealed that GPx3 was co-localized with UCHL1 (Figure 1I), a marker of human SSCs, in the human testicular tissue. Taken together, these results indicate that GPx3 is expressed in human SSC line and human SSCs.

### Human SSC line expresses a series of the specific genes and proteins of human SSCs

To explore the role of GPx3 in regulating the fate decisions of human SSCs, human SSC line was employed in this study since it possesses the characteristics of the primary human SSCs and can be proliferated and expanded *in vitro* (Hou et al., 2015). For the identification of the human SSC line, RT-PCR revealed that a number of genes, including *GPR125*, *GFRA1*, *UCHL1*, *MAGEA4*, *THY1*, *RET*, and *PLZF,* were detected in the human SSC line (Figure 1A), whereas no cDNA was utilized as the negative control (Figure 1A). Meanwhile, immunocytochemistry illustrated that GPR125 (Figure 1B), UCHL1 (Figure 1C), GFRA1 (Figure 1D), THY1 (Figure 1E), and PLZF (Figure 1G) are expressed in the human SSC line. It is worth noting that overexpression of *SV40* mRNA and SV40 proteins was detected in the human SSC line (Figures 1A, F), as previously described (Hou et al., 2015). Considered together, these data implicate that the human SSC line utilized in the study is human SSCs phenotypically.

### GPx3 knockdown results in a decrease in proliferation, DNA synthesis, and the expression levels of cyclin B1 and cyclin-dependent kinase 2 (CDK2) proteins as well as the enhancement of apoptosis of the human SSC line

**FIGURE 2:**
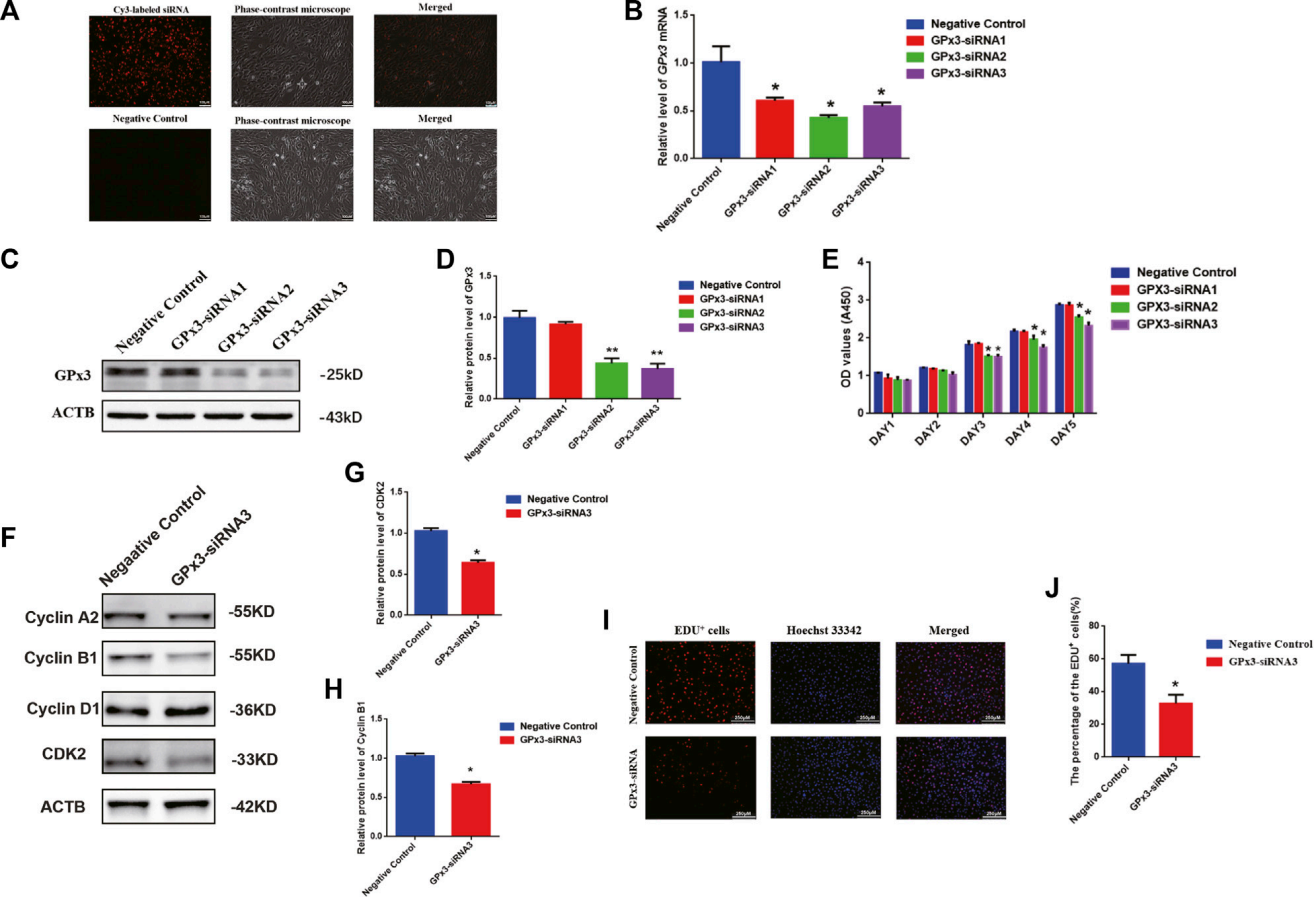
GPx3 knockdown inhibited proliferation, DNA synthesis, and cyclin B1 and CDK2 expression levels in the human SSC line: **(A)** The transfection efficiency of GPx3-siRNA was measured by Cy3-labeled siRNA oligonucleotides in the human SSC line using a fluorescence microscope. Scale bars in A = 100 μm. **(B)** Real-time PCR displayed the level of *GPx3* mRNA in the human SSC line with the transfection of control siRNA and GPx3-siRNA1, 2, and 3. **(C–D)** Western blots showed the changes in the GPx3 protein in the human SSC line with the treatment of control siRNA and GPx3-siRNA1, 2, and 3. **(E)** CCK-8 assay demonstrated the capability of the proliferation in the human SSC line without or with the treatment of GPx3-siRNA1-3. **(F–H)** Western blotting exhibited the level of changes in cyclin A2, B1, D1, and CDK2 proteins in the human SSC line 72 h after transfection of control siRNA and GPx3-siRNA3. **(I–J)** An EDU incorporation assay revealed the percentages of the EDU-positive cells influenced by control siRNA and GPx3-siRNA3.

To probe the biological function of GPx3 in mediating the human SSC line, RNA interference (RNAi) was applied, and three pairs of small interfering RNAs (siRNA) that targeted diverse regions of *GPx3* mRNA were designed by GenePharma (China) to guarantee the efficiency of gene knockdown. As the negative control, the control siRNA was designed with no targeting *GPx3* sequence. To assess the transfection efficiency of siRNAs, cyanine 3 (Cy3)-labeled siRNA oligonucleotides were used to demonstrate that the transfection efficiency of siRNAs in the human SSC line was over 85% (Figure 2A, upper panel). Real-time PCR further revealed that, compared to the control siRNA (negative control), the relative level of *GPx3* mRNA was notably decreased in the human SSC line 24 h after transfection with *GPx3* siRNAs (Figure 2B). Western blotting displayed that the protein level of GPx3 was diminished evidently in the human SSC line transfected with GPx3-siRNA2 and GPx3-siRNA3 after 60 h (Figures 2C,D). These data illustrate that GPx3-siRNA2 and GPx3-siRNA3 can effectively knockdown GPx3 of the human SSC line. The CCK-8 assay showed that GPx3-siRNA2 and GPx3-siRNA3 inhibited the proliferation of the human SSC line 72–120 h after transfection, while the inhibitory effect of GPx3-siRNA3 was the most prominent (Figure 2E). In order to guarantee that the human SSC line remained the same phenotype after transfection with GPx3-siRNAs, immunocytochemistry was performed and showed that GPR125, GFRA1, and UCHL1, the biomarkers of human SSCs, were expressed stably in the human SSC line, which reflected that the human SSC line treated with GPx3-siRNAs was still human SSCs phenotypically (Supplementary Figure S1B). Furthermore, compared to the control siRNA, EDU-positive cells were decreased in the human SSC line after transfection with GPx3-siRNA3 (Figures 2I,J). Collectively, these results imply that GPx3 knockdown inhibits proliferation and DNA synthesis of the human SSC line. In addition, we examined the expression changes of a series of cell cycle proteins, including cyclin A2, cyclin B1, cyclin D1, and CDK2. GPx3-siRNA3 inhibited the expression levels of cyclin B1 and CDK2 in the human SSC line 72 h after treatment (Figures 2F–H), whereas there was no obvious change in the levels of cyclin A2 and cyclin D1 proteins in the human SSC line without or with GPx3-siRNA3 treatment (Figures 2F–H).

**FIGURE 3:**
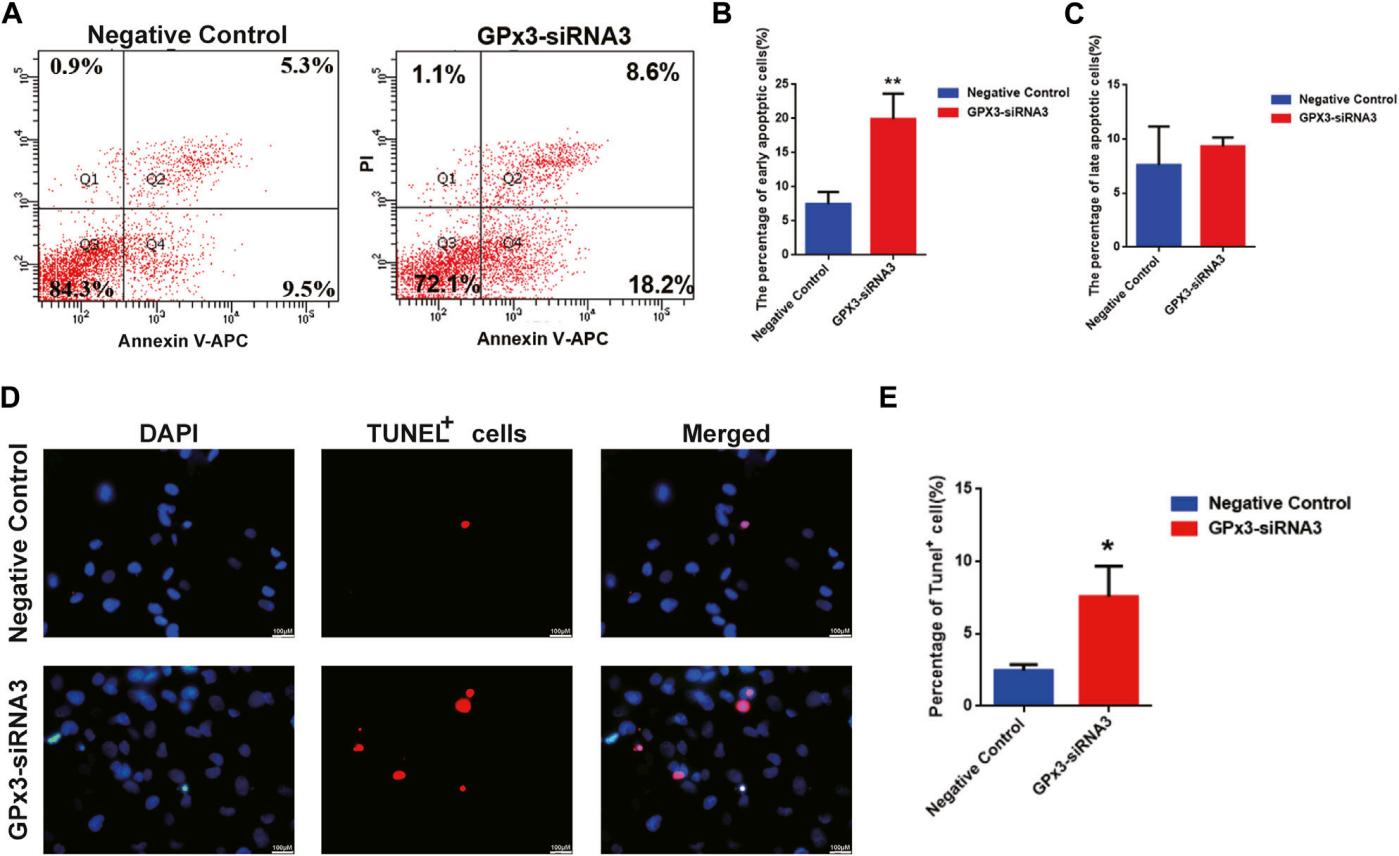
GPx3 knockdown enhanced the percentages of apoptotic cells in the human SSC line. **(A–C)** Annexin V/propidium iodide (PI) staining and flow cytometry were utilized to determine the percentages of the early and late apoptosis of the human SSC line with control siRNA and GPx3-siRNA3 for 3 days. **(D–E)** A TUNEL assay showed the apoptosis of the human SSC line with control siRNA and GPx3-siRNA3. Scale bars in D = 100 μm.

In order to assess the influence of GPx3 on mediating the apoptosis of the human SSC line, annexin V/propidium iodide (PI) staining and flow cytometry were employed and showed that the percentages of the early, but not late, apoptosis of the human SSC line were increased by GPx3-siRNA3 transfection at 72 h (Figures 3A–C). A TUNEL assay displayed that GPx3-siRNA3 significantly increased the percentages of TUNEL-positive cells in the human SSC line compared to the control siRNA (Figures 3C,D). Considered together, these findings suggest that knockdown of GPx3 enhances early apoptosis in the human SSC line.

### GPx3 mediates the proliferation and apoptosis of the human SSC line via CXCL10

**FIGURE 4:**
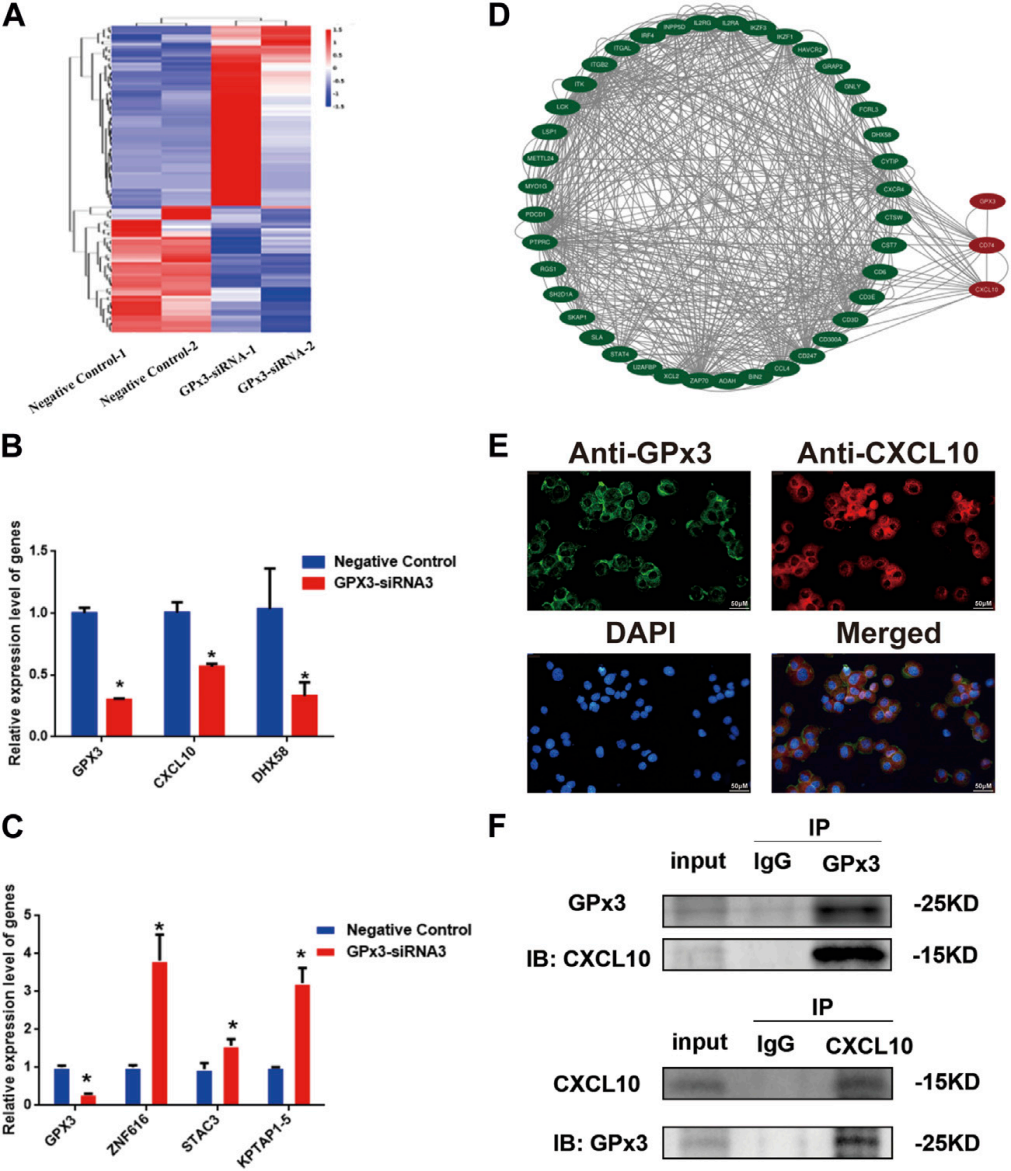
CXCL10 was a downstream target of GPx3 in the human SSC line. **(A)** Hierarchical clustering illustrated the differentially expressed genes (DEGs) between GPx3-siRNA3 and control siRNA by RNA sequencing. **(B–C)** Real-time PCR demonstrated the changes in *CXCL10*, *DHX58*, *GPx3*, *ZNF616*, *STAC3*, and *KPTAP1-5* mRNA influenced by GPx3-siRNA3 compared to the control siRNA. **(D)** Protein–protein interactions (PPIs) predicted that there was an interaction between GPx3 and CXCL10. **(E)** Double immunostaining displayed the cellular localization of GPx3 and CXCL10 in the human SSC line. Scale bars in E = 50 μm. **(F)** Co-IP revealed that there was an interaction between GPx3 and CXCL10 proteins in the human SSC line.

To uncover the molecular mechanism of GPx3 controlling the proliferation and apoptosis of the human SSC line, we screened the changes in the transcriptional profiles of the human SSC line with transfection of GPx3-siRNA3 and control siRNA to identify targets of GPx3 utilizing RNA sequencing (Figure 4A). There were approximately 15,000 genes present in the human SSC line, while 64 genes were upregulated and 45 genes were downregulated by GPx3-siRNA3. To ensure the accuracy of the RNA sequencing data, several differentially expressed genes (DEGs) with distinct changes were selected and verified by qPCR, including *CXCL10*, *DHX58*, *ZNF616*, *STAC3*, and *KPTAP1-5.* Our qPCR analysis showed that the levels of *CXCL10*, *DHX58*, and *GPx3* mRNA were decreased by GPx3-siRNA3 (Figure 4B), whereas the transcript levels of *ZNF616*, *STAC3*, and *KPTAP1–5* were increased by GPx3-siRNA3 (Figure 4C), which was consistent with our RNA sequencing data. Notably, *CXCL10* mRNA was stably decreased by GPx3-siRNA3. Meanwhile, both the PPI (protein–protein interaction) analysis and STRING database predicted that there was an association between GPx3 and CXCL10 (Figure 4D). To determine whether there was an interaction between GPx3 and CXCL10, double immunostaining was conducted for GPx3 and CXCL10 in the human SSC line and testicular tissues. We revealed that GPx3 was co-localized with CXCL10 in both the human SSC line and human testicular tissue (Figure 4E; Supplementary Figure S2). Interestingly, co-immunoprecipitation (co-IP) displayed that CXCL10 was pulled down by GPx3 in the human SSC line (Figure 4F, upper panel) and that CXCL10 could bind to GPx3 in these cells (Figure 4F, lower panel). Collectively, these data imply that GPx3 interacts with CXCL10 in the human SSC line.

### CXCL10 knockdown suppresses proliferation and promotes the apoptosis of the human SSC line

**FIGURE 5:**
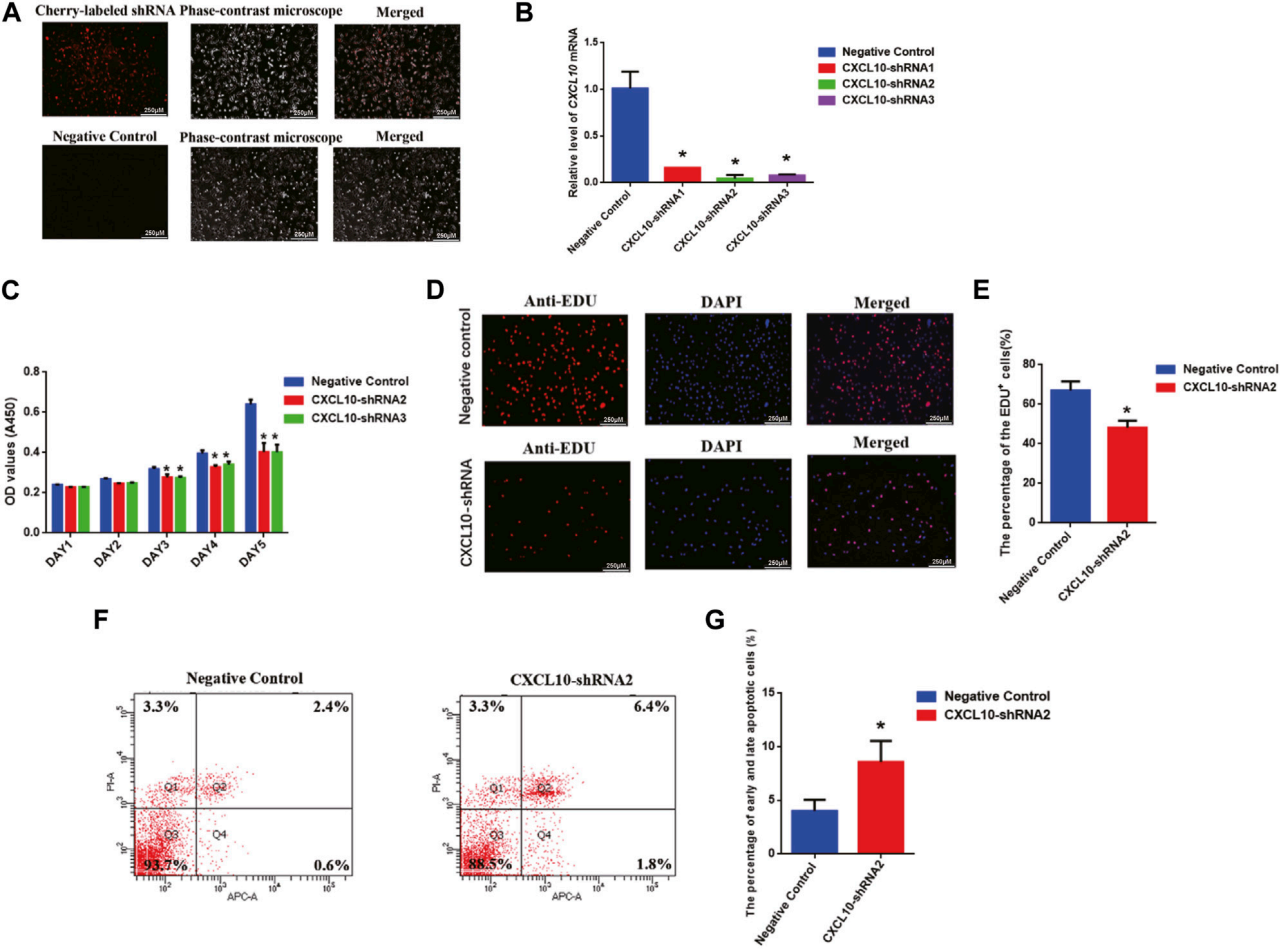
Effect of CXCL10 knockdown on the proliferation, DNA synthesis, and apoptosis of the human SSC line: **(A)** The fluorescence microscope was utilized to detect the transfection efficiency through cherry-labeled CXCL10-shRNA. Scale bars in A = 250 µm **(B)** The knockdown efficiency of CXCL10 by shRNA was detected by qPCR. **(C)** A CCK-8 assay demonstrated the proliferation ability of the human SSC line between the treatment with CXCL10-shRNA and control shRNA. **(D–E)** An EDU incorporation assay showed the percentages of the EDU-positive cells in the human SSC line treated with CXCL10-shRNA and control shRNA. Scale bars in D = 250 μm. **(F–G)** Annexin V/PI staining and flow cytometry revealed the percentages of apoptotic cells in the human SSC line affected by CXCL10-shRNA2 and control shRNA.

To further unveil the function of CXCL10 in regulating the proliferation and apoptosis of the human SSC line, we established the stable CXCL10 knockdown human SSC line by CXCL10-shRNA. Three pairs of the CXCL10 short-hairpin RNAs (shRNA), i.e., CXCL10-shRNA 1, 2, and 3, were designed to ensure the efficiency of knockdown, while the control shRNA was used as the negative control. Cherry-labeled shRNA exhibited that the transfection efficiency was over 80% (Figure 5A). To confirm that the human SSC line after the transfection of CXCL10-shRNAs still retained the characteristics of human SSCs, immunocytochemistry was utilized and revealed that some biomarkers of human SSCs, including GPR125, GFRA1, and UCHL1, were detected in the human SSC line, suggesting that the human SSC line transfected with CXCL10-shRNAs were human SSCs phenotypically (Supplementary Figure S1C). Real-time PCR showed that the level of *CXCL10* mRNA was decreased by CXCL10-shRNA 1, 2, and 3 in the human SSC line (Figure 5B). A CCK-8 assay revealed that both CXCL10-shRNA2 and CXCL10-shRNA3 suppressed the proliferation of the human SSC line from 72 to 120 h (Figure 5C). EDU analysis demonstrated that the percentage of the EDU-positive cells was diminished in the human SSC line by CXCL10-shRNA2 compared with the control shRNA (Figures 5D,E). Taken together, these data suggest that CXCL10 knockdown inhibits the proliferation and DNA synthesis of the human SSC line. In contrast, flow cytometry analysis indicated that the number of early and late apoptotic cells was significantly increased in the human SSC line by CXCL10-shRNA2 compared with the control shRNA (Figures 5F,G). Considered together, CXCL10 knockdown enhances the apoptosis of the human SSC line.

## Materials and methods

### Human testicular tissues

Human testicular tissues utilized in this study were obtained from patients diagnosed with obstructive azoospermia (OA) in the Hunan Cancer Hospital. This study was approved by the Ethics Review Committee of Hunan Normal University.

### Human SSC line culture, transfection, and generation of the stable CXCL10 knockdown cell line

The human SSC line established at our laboratory by transfecting with lenti-EF1α-SV40LargeT-IRES-eGFP was used in this study (Hou et al., 2015). The cell line was cultured in DMEM/F12 supplemented with 10% fetal bovine serum (Gibco) and 1% penicillin–streptomycin at 34°C in an incubator with 5% CO_2_. The cells were passaged every 3 days with 0.53 mmol/L EDTA and 0.5 g/L trypsin.

To explore the function of GPx3 in regulating the fate decisions of human SSCs, three different siRNAs targeting *GPx3* were synthesized from GenePharma (China), while the siRNA targeting no sequence of the *GPx3* gene was used as the negative control. The cherry-labeled siRNA oligonucleotides were utilized to assess the transfection efficiency of the siRNAs, and the GPx3-siRNA sequences were listed in Supplementary Table S1. The siRNA transfection was conducted using 20 μM GPx3-siRNAs or the negative control group via the transfection agent Lipofectamine 3000 (Thermo Fisher, United States) according to the manufacturer’s protocol. After 48 h of the transfection, the cells were dissociated to detect the changes in numerous genes and proteins.

To generate the CXCL10 stable knockdown cell line, 293T cells were utilized to produce the lentivirus using the ViraPower kit (Invitrogen, Carlsbad, CA) according to the manufacturer’s protocol. Three different clones were designed by Gene Corporation (China). To ensure the transfection efficiency, puromycin was used to select the cells in which the CXCL10-shRNAs were packaged, and the transfection efficiency was determined by quantitative real-time PCR.

### Immunocytochemistry and immunohistochemistry

Cells were placed onto the slide glass by centrifuging and fixed with 4% paraformaldehyde (PFA) at room temperature for 15 min, and they were blocked by 5% BSA (Sigma) for 60 min. Primary antibodies were diluted by 5% BSA and utilized to be incubated with cells at 4°C overnight. After washing with PBS (phosphate-buffered saline), the cells were incubated with secondary antibodies at room temperature for 60 min. Moreover, 4,6-diamino-2-phenyl indole (DAPI) was used to stain the nuclei of the cells. The images were captured using a fluorescence microscope (Leica, United States).

Testicular tissue sections were deparaffinized with turpentine and rehydrated, and antigen retrieval was accomplished with 1x sodium citrate buffer reagent by heating for 20 min using a microwave oven. Sections were blocked with 5% BSA and 0.1% Triton X-100 for 30 min at room temperature and incubated with primary antibodies at 4°C overnight. After washing four times with PBS-T (phosphate-buffered saline with addition of 0.05% Tween-20), testicular tissue sections were incubated with secondary antibodies for 60 min at room temperature. Cell nuclei were stained with DAPI for 5 min at room temperature. After washing three times with PBS and once with distilled water, anti-fade mounting medium was used to seal the slides, and images were obtained using a fluorescence microscope (Leica, United States). The detailed information of antibodies used for immunocytochemistry and immunohistochemistry, including the sources of the vendors and the working dilution of each antibody, was shown in Supplementary Table S2.

### RNA extraction, reverse transcription polymerase chain reaction (RT-PCR), and quantitative real-time polymerase chain reaction (qPCR)

To evaluate the quality and purity of total RNA, the NanoDrop 2000 (Thermo Fisher) was utilized, and the ratio of A_260_/A_280_ was 1.8–2.0, which qualified for RT-PCR and qPCR. RT was conducted using the One-Step cDNA Synthesis SuperMix (TransGen Biotech, China) and the MiniAmp Thermal Cycler (Thermo Fisher) to convert the total RNA into cDNA. The PCR reaction was performed as follows: 95°C for 5 min; denaturation at 95°C for 30 s, annealing at 52°C–60°C for 30 s, and elongation at 72°C for 45. The PCR products were incubated at 72°C for 7 min. The primers of genes, including *ACTB*, *GPR125*, *GFRA1*, *UCHL1*, *MAGEA4*, *CD90*, *SV40*, *VASA*, *RET*, *PLZF*, and *GPx3*, were designed and were listed in Supplementary Table S3. The 2% agarose gels were used to separate the PCR products through electrophoresis, and PCR products with ethidium bromide could be visualized by Image Analysis System ChampGel 5,000 (Sage Creation, China).

The qPCR was conducted with cDNA as previously described using the SYBR Green Premix Pro Taq HS qPCR Kit on a CFX Connect Real-Time System (Bio-Rad, United States). The data were analyzed through the ΔΔCt method, and the reference gene was *ACTB*. The primers of the real-time PCR were listed in Supplementary Table S4.

### Western blots

The protein of the human SSC line transfected with the GPx3-siRNA1, GPx3-siRNA2, GPx3-siRNA3 or CXCL10-shRNA1, CXCL10-shRNA2, and CXCL10-shRNA3 was extracted by the protein lysate, which was composed of RIPA (Beyotime, China), 10 μL/mL PMSF, and 10 μL/mL protease inhibitor. The cell lysate was centrifuged at 12,000 rpm for 20 min to eliminate cell debris according to the manufacturer’s instruction. The supernatant was collected to measure protein concentrations using a bicinchoninic acid (BCA) kit (Dingguo, China). Thirty micrograms of proteins were used for 10%–14% SDS-PAGE (sodium dodecyl sulfate-polyacrylamide gel electrophoresis). The proteins were transferred onto PVDF (polyvinylidene fluoride) membranes using the wet transfer method, and blocking buffer was utilized at room temperature for 60 min. The membrane was incubated with the primary antibodies at 4°C overnight. The primary antibodies utilized in this study included ACTB (Cell Signaling Technology, United States, catalog no. 3700s, 1:1,000), GPx3 (ABclonal, China, catalog no. A12596, 1:500), cyclin A2 (Cell Signaling Technology, United States, catalog no. 4656T, 1:1,000), cyclin B1 (Cell Signaling Technology, United States, catalog no. 4138T, 1:1,000), cyclin D1 (Cell Signaling Technology, United States, catalog no. 2978T, 1:1,000), and CDK2 (Cell Signaling Technology, United States, catalog no. 2546s, 1:1,000). Next, the membrane was washed with TBST (tris-buffered saline containing 0.1% Tween-20) three times, and the horseradish peroxidase (HRP)-labeled secondary antibody was used at room temperature for 60 min and followed by washing with TBST three times. Subsequently, an enhanced chemiluminescence (Beyotime, China) kit was employed to detect proteins using the Mini Chemiluminescent Imaging and Analysis System (Sage Creation, China).

### Cell counting kit-8 (CCK-8) assay

The CCK-8 assay was employed to detect the proliferation ability of the human SSC line by GPx3-siRNAs and CXCL10-shRNAs. The human SSC line was plated onto 96-well microtiter plates, and a density of 2 × 10^3^ human SSCs per well was used. The cells were transfected with the GPx3-siRNAs, CXCL10-shRNAs, control siRNA, or control shRNA. Cell culture medium was substituted with the 10% CCK-8 reagent, and the absorbance was measured by a multi-mode microplate reader (BioTek, Germany) at 450 nm after incubation for 4 h. The CCK-8 assay was performed for 5 days.

### EDU incorporation assay

Human SSC line treated with the GPx3-siRNA3 or CXCL10-shRNA was placed onto the 96-well microtiter plates at a density of 3,000 cells per well. EDU reagent (RiboBio, China) was added into the 96-well microtiter plates and incubated for 12 h. Next, 4% PFA was utilized to fix the cells for 30 min at room temperature. Then, 2 mg/ml glycine was added to the cells to neutralize for 5 min, and 0.5% Triton X-100 (Sigma, United States) in PBS was used to permeabilize for 10 min at room temperature. The Apollo staining reaction solution was prepared and utilized to stain the cells for 30 min at room temperature in the dark. Finally, cell nuclei were stained with Hoechst 33342 or DAPI for 10 min, and the number of EDU-positive cells was calculated from at least 500 cells using a fluorescence microscope (Leica, Germany).

### Annexin V and propidium iodide staining and flow cytometry analysis

To detect the apoptosis of the human SSC line affected by GPx3, the cells after transfection without or with GPx3-siRNA3 and CXCL10-shRNA2 for 72 h were digested with trypsin without EDTA. The cells were obtained by centrifugation, and then 100 μL Annexin V-binding buffer was added into cells after washing twice with the pre-cooleding PBS. Meanwhile, the cells were stained with 5 μL APC annexin V and 10 μL propidium iodide solution (BioLegend, United Kingdom) and incubated for 15 min in the dark. The percentages of the apoptosis cells were analyzed by flow cytometry (BD Biosciences, NJ, United States).

### TUNEL assay

The human SSC line was treated with GPx3-siRNA3 or control siRNA. The TMR TUNEL Cell Apoptosis Detection Kit (Servicebio, G1502) was employed to measure cell apoptosis according to the manufacturer’s instruction. These cells were incubated with 20 μg/mL of proteinase K for 10 min at room temperature. Next, TMR-5-dUTP Labeling Mix/TdT enzyme buffer was added to mark apoptotic cells. Nuclei of cells were stained with DAPI, and images were captured by fluorescence microscopy (Leica, DMi8, Germany).

### RNA sequencing

After the transfection of GPx3-siRNA3 for 36 h, the human SSC line was washed twice with PBS and lysed by TRIzol (TaKaRa). Subsequently, cell lysates were frozen rapidly in liquid nitrogen and sent to OE biotech for RNA sequencing. The libraries were sequenced on an Illumina HiSeq X Ten platform, and 150 bp paired-end reads were generated. DEG analysis was performed using the DESeq (2012) R package. The standards for DEGs were *p*-value < 0.05 and fold change ≥2 or ≤-2. 

### Co-immunoprecipitation (co-IP)

The interactions between GPx3 and CXCL10 proteins were determined using a Co-IP kit (Thermo). IP lysis buffer was utilized to digest and lyse the human SSC line for 10 min. The lysate was centrifuged for 20 min at 12,000 rpm, and the supernatant was retained. The IP antibodies were added to the supernatant, including GPx3 or CXCL10, and it was incubated at 4°C overnight to ensure the formation of the complex. The magnetic A/G beads were employed to absorb the complex by incubating at room temperature for 1 h. The protein interactions were detected through Western blots using antibodies against GPx3 or CXCL10.

### Statistical analysis

All data were obtained from three or more experiments and presented as mean ± SEM. The statistical analysis was performed by GraphPad Prism 6.01, and credibility was examined through the unpaired *t*-test. *P*< 0.05 indicated a statistically significant difference.

## Discussion

The molecular mechanisms regulating the fate determinations of human SSCs remain largely unknown. In this study, we have elucidated, for the first time, the expression, function, and downstream target of GPx3 in controlling the proliferation, DNA synthesis, and apoptosis of the human SSC line. We found that GPx3 was present in the cytoplasm of male germ cells and that it was highly expressed in the human SSC line and SSCs in the human testicular tissues.

We utilized RNA interference (RNAi) to explore the function and mechanism of GPx3 in the human SSC line. RT-PCR and immunocytochemistry were performed to detect a series of biomarkers of human SSCs, and the results illustrated that the human SSC line we utilized in this study assumed the phenotypic characteristics of the human primary SSCs. Meanwhile, the high level of SV40 expression verified that the human SSC line could be cultured and expanded *in vitro*. We have previously found that PAK1 regulates the proliferation and apoptosis of the human SSC line (Fu et al., 2018). After PAK1 has been silenced by siRNAs, the capabilities of the proliferation, DNA synthesis, and anti-apoptosis are obviously decreased. RNA sequencing indicate that PAK1 knockdown leads to the reduction in GPx3 in the human SSC line. GPx3 has been reported as an important regulator for numerous cancers, e.g., lung cancer (An et al., 2018), esophageal carcinoma (Zhu et al., 2018), melanoma (Chen et al., 2016; Yi et al., 2019), prostate cancer (Chang et al., 2014; Chang et al., 2016; Chang et al., 2018), hepatocellular carcinoma (Qi et al., 2014), bladder cancer (Song et al., 2020), and ovarian cancer (Worley et al., 2019). For non-small-cell lung cancer, microRNA-196a manipulates the expression of GPx3 to inhibit the self-renewal ability of stem cells (Liu et al., 2019). Nonetheless, the molecular mechanism of GPx3 in mediating human spermatogenesis remains unclear. In this study, we first explored the function of GPx3 in manipulating the proliferation, DNA synthesis, and apoptosis of the human SSC line. We found that GPx3 silencing led to a decrease in the abilities of proliferation and DNA synthesis as well as an increase in the early apoptosis of the human SSC line.

In order to explore the molecular mechanism of GPx3 in regulating the fate decisions of human SSCs, RNA sequencing was performed to identify the downstream target of GPx3. Notably, the expression level of *CXCL10* mRNA was remarkably decreased when GPx3 was silenced in the human SSC line. Moreover, our double immunostaining, co-IP, PPI (protein–protein interaction) analysis, and the STRING database indicated that there was an interaction between GPx3 and CXCL10 in these cells. To ensure the reliability of RNA-seq, qPCR displayed that *CXCL10* mRNA was greatly decreased when GPx3 was silenced by GPx3-siRNA3. CXCL10, known as interferon γ-induced protein 10 (IP-10), has been reported in multitudinous tumors and autoimmune diseases, including breast carcinoma (Wu et al., 2020), melanoma (Feldman et al., 2002), sarcoma (Sun et al., 2005), and colon cancer (Cambien et al., 2009). In autoimmune prostatitis, the inhibition of CXCL10 expression diminishes the secretion of inflammatory mediators of macrophages via CXCR3-mediated ERK and p38 MAPK activation (Hua et al., 2021). Meanwhile CXCL10 facilitates the tamoxifen resistance of breast cancer cells to enable the prognosis of breast carcinoma via the AKT pathway (Wu et al., 2020). Furthermore, overexpression of CXCL10 inhibits tumor growth in melanoma and sarcoma (Feldman et al., 2002; Sun et al., 2005). Nevertheless, the function of CXCL10 in controlling human SSCs remains to be clarified. The stable CXCL10 knockdown cell line was established by transfecting CXCL10-shRNA into the human SSC line via lentivirus-mediated transfection. Our CCK-8 and EDU assays TUNEL assay, and flow cytometry analysis implicated that CXCL10 silencing suppressed the proliferation of the human SSC line and enhanced the apoptosis of these cells.

In summary, we have demonstrated, for the first time, that GPx3 is expressed in human SSCs and that GPx3 knockdown suppresses the proliferation and DNA synthesis of the human SSC line and enhances the early apoptosis of these cells. We have also revealed that GPx3 mediates the target CXCL10 whose silencing leads to a decrease in the proliferation of the human SSC line and an increase in the apoptosis of human SSCs. This study, thus, provides a novel insight into molecular mechanisms underlying the fate decisions of the human SSCs and human spermatogenesis.

## Data Availability

The datasets presented in this study can be found in online repositories. The names of the repository/repositories and accession number(s) can be found below: NCBI BioProject at PRJNA975339.
