# Supplementary material for: GPx3 knockdown inhibits the proliferation and DNA synthesis and enhances the early apoptosis of human spermatogonial stem cells via mediating CXCL10 and cyclin B1
Source: Front Cell Dev Biol. 2023 Jul 7;11:1213684. doi: 10.3389/fcell.2023.1213684 (PMC10361659; doi:10.3389/fcell.2023.1213684)
Supplement: Supplementary file 1 [file DataSheet1.pdf]

Supplementary Tables 1-4 and Figures 1-2

Table S1. The Sequences for GPx3-siRNAs

**siRNA Names**	**The Sequences of siRNAs**
GPx3-siRNA1	**sense:** 5’-GGCAUAAGUGGCACCAUUUTT-3’<br>**antisense:** 5’-AAAUGGUGCCACUUACGCCTT-3’
GPx3-siRNA2	**sense:** 5’-CCUUCCUACCCUCAAGUACTT-3’<br>**antisense:** 5’-AUACUUGAGGGUAGGAAGGTT-3’
GPx3-siRNA3	**sense:** 5’-CCAUGAAGGUUCACGACAUTT-3’<br>**antisense:** 5’-AUGUCGUGAACCUUCAUGGTT-3’

Table S2. The Detailed Information on Primary Antibodies Used for Immunocytochemistry

Antibodies	Vendors	Sources	Working Dilutions
GPR125	Abcam	rabbit	ICC: 1:200
UCHL1	AbDSerotec	Mouse	ICC: 1:200
GFRA1	Abcam	Goat	ICC: 1:200’
THY1	Abcam	Rabbit	ICC: 1:200
SV40	Santa Cruz Biotechnology	Mouse	ICC: 1:200
PLZF	Santa Cruz Biotechnology	Rabbit	ICC: 1:200

Table S3. The Sequences of the Primers of Genes for RT-PCR

Gene Names	Gene Accession Numbers	Primer sequences (5'-3')	Product sizes (bp)
ACTB	NC_000007.14	**forward:** AGAGCTACGAGCTGCCTGAC<br>**reverse:** AGCACTGTGTTGGCGTACAG	184
GPR125	NC_000004.12	**forward：** TGGGTAGGAGTGACAGCTCG<br>**reverse:** TTGGCCGACTGCCGTAATTC	190
GFRA1	NC_000010.11	**forward：** ACCAAGTACCGCACGCTAAG<br>**reverse:** TATGGGGAATCCTCCAGCAGA	233
UCHL1	NC_000004.12	**forward :** AGCTGAAGGGACAAGAAGTTAG<br>**reverse:** TTGTCATCTACCCGACATTGG	265
MAGEA4	NC_000023.11	**forward：** CTTACCCACTACCATCAGCTTC<br>**reverse：** TGATGACTCTCTCCAGCATTTC	212
THY1	NC_000023.11	**forward：** CAGAAGGTGACCAGCCTAAC<br>**reverse:** TTGTCATCTACCCGACATTGG	233
SV40		**forward：** GAACAGCCCAGCCACTATAA<br>**reverse:** ACTCCAGCCATCCATTCTTC	248

VASA	NC_000005.10	**forward:** CATACTTGCAGGACGAGATTTG<br>**reverse:** CAACTCTTTAAAACGACTGGCA	127
RET	NC_000010.11	**forward:** ATGAGAACAACTGGATCTGCAT<br>**reverse:** GAAGAAGGAGAAGTATACGCGG	209
PLZF	NC_000011.10	**forward:** GGACAAGGTTGAGGAAAGAGG<br>**reverse:** CAACACGGAGTAGATGCCCAG	205
GPx3	NC_000005.10	**forward:** GAGCTTGCACCATTCGGTCT<br>**reverse:** GGGTAGGAAGGATCTCTGAGTTC	94

Table S4. The Sequences of Gene Primers for qPCR

Gene Names	Primer Sequences （5’-3’)	Tm (℃)
GPx3	**forward**：GAGCTTGCACCATTCGGTCT<br>**reverse:** GGGTAGGAAGGATCTCTGAGTTC	63
CXCL10	**forward**：CAAATCTGCTTTTTAAAGAATGCTC<br>**reverse:** AAGAATTTGGGCCCCTTG	52
DHX58	**forward**：GGAAGTGATCTTACCTGCTCTGG<br>**reverse:** TTGCCTCTGTCTACCGTCTCT	57
ZNF616	**forward**：CAGTGACCGTTCAGCTTTTGC<br>**reverse:** CGATGCCCTACAAGATGTGAAT	52
STAC3	**forward**：CCTAAGCTGGTCAACGATAAGC<br>**reverse:** CATAGGACTGACAGTGTTCATGG	50
KPTAP1-5	**forward**：CTCCATCAGTGGGACCTGTG<br>**reverse:** CAGTTCCGCAGGAGCTGAT	60

Figure S1.

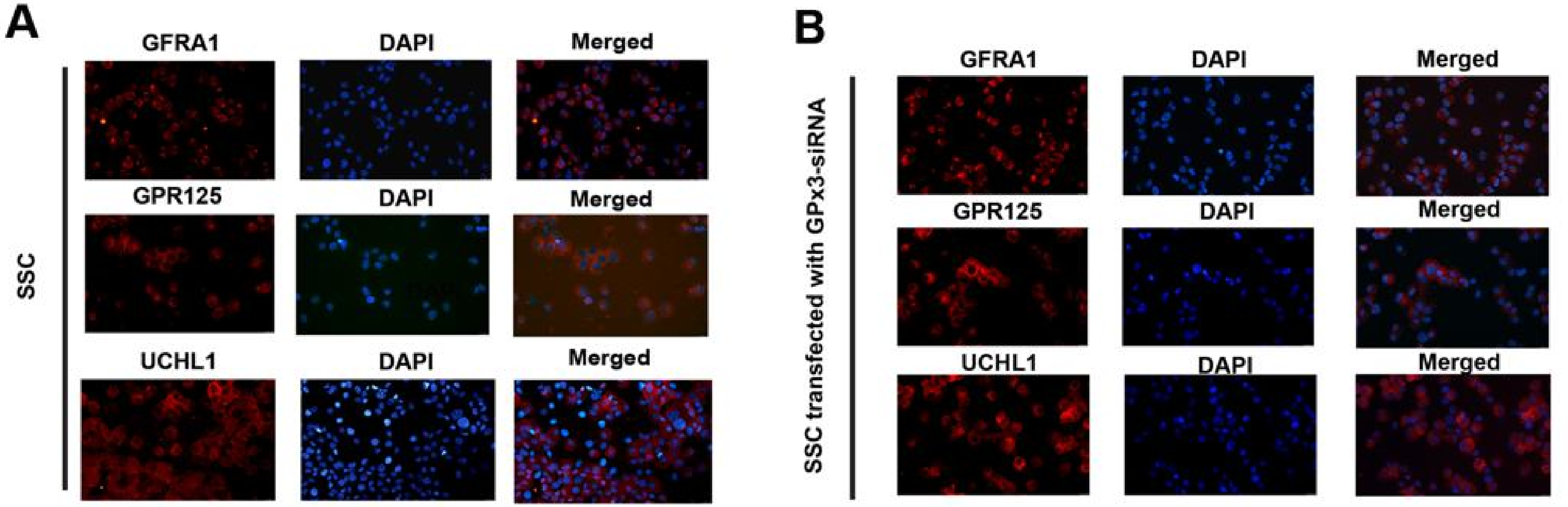
A
GFRA1
DAPI
Merged
GPR125
DAPI
Merged
UCHL1
DAPI
Merged
SSC
B
GFRA1
DAPI
Merged
GPR125
DAPI
Merged
UCHL1
DAPI
Merged
SSC transfected with GPx3-siRNA

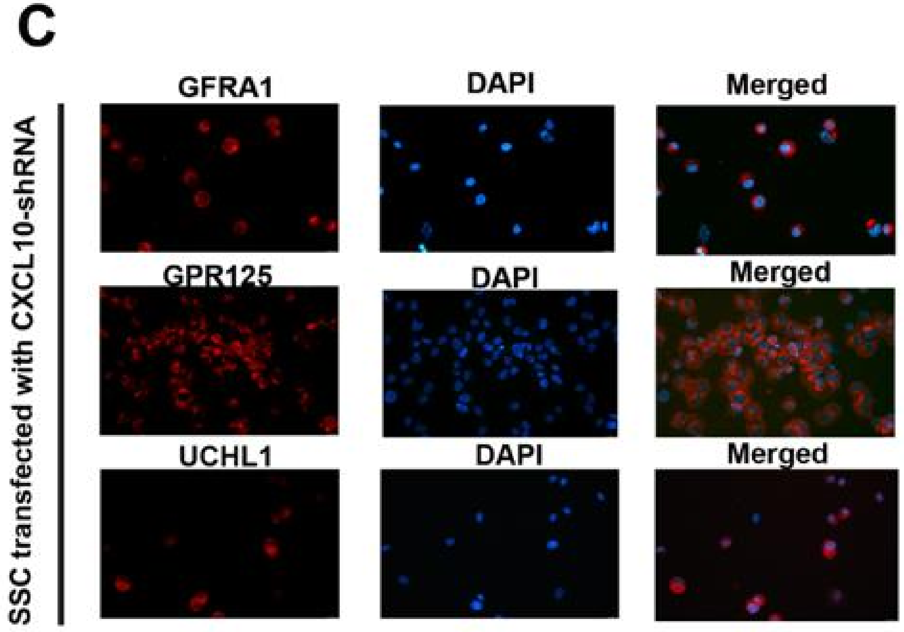

Figure S1. Identification of the human SSC line and human SSC line after transfection of GPx3-siRNAs or CXCL10-shRNA. (A) Immunocytochemical displayed that the expression of several proteins in the human SSC line, including GPR125, GFRA1, and UCHL1. (B-C) Immunocytochemical revealed that the markers of the human SSCs were still expressed in the human SSC line after transfection of GPx3-siRNAs and CXCL10-shRNAs, including GPR125, GFRA1, and UCHL1.

Figure S2.

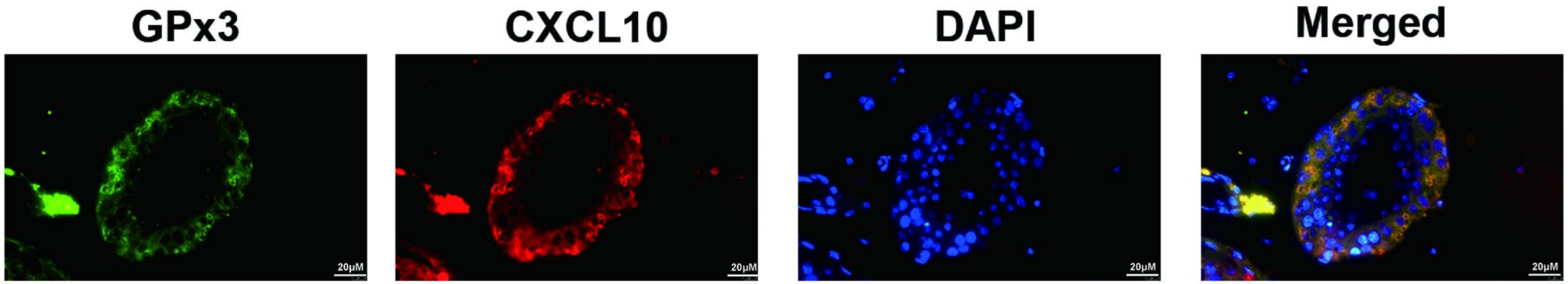

Figure S2. The colocaliztion of GPx3 and CXCL10 in human testicular tissues. Immunohistochemistry by double staining showed that cellular location of GPx3 and CXCL10 proteins in human testis. Scale bars: 20 μm.
